# Supplementary material for: Comparative efficacy of intraoperative radiotherapy and external boost irradiation in early-stage breast cancer: a systematic review and meta-analysis
Source: PeerJ. 2023 Sep 18;11:e15949. doi: 10.7717/peerj.15949 (PMC10512934; doi:10.7717/peerj.15949)
Supplement: Supplemental Information 3 [file peerj-11-15949-s003.docx]

1.The rationale for conducting the systematic review / meta-analysis

Answer: In the past 10 years, multiple randomized controlled trials (RCTs) have been conducted to evaluate the efficacy of intraoperative IORT and postoperative EBRT on reducing LR, preventing distant metastasis, and prolonging DFS and OS in early-stage breast cancer patients [6, 7]. However, due to the diversity of demographics, histopathology, and systemic treatment patterns in different clinical trials, the comparative effect of these two therapies remains controversial [8, 9]. Thus, we have conducted this systematic review and meta-analysis to critically compare the efficacy of IORT and EBRT for the treatment of early-stage breast cancer, so as to provide evidence-based support for clinical decision-making.

2.The contribution that it makes to knowledge in light of previously published related reports, including other meta-analyses and systematic reviews.

Firstly, we included RCTs and propensity-matching scoring subgroup study of high-quality. Secondly, we assessed the short-term (5-year) and long-term (15-year) follow-up outcomes. Thirdly, our meta-analysis involved a large sample size, and the participants covered multiple centers in various regions so that avoided possible racial and social impacts to a large extent. We have conducted this systematic review and meta-analysis to critically compare the efficacy of IORT and EBRT for the treatment of early-stage breast cancer, so as to provide evidence-based support for clinical decision-making.and get the conclusion：IORT and EBRT have similar risk of short-term LR in patients with early-stage breast cancer, and their effect on long-term LR remains elusive. No significant differences are observed between these two methods in reducing distant metastasis risk and improving DFS and OS in patients with early-stage breast cancer. IORT would be more convenient, time-saving, and cost-effective, and would be more effective in reducing the side effects and toxicity. However, these advantages must be weighed against the possibility of increasing the risk of long-term LR.
